# Sterile Alpha Motif Containing 7 (Samd7) Is a Novel Crx-Regulated Transcriptional Repressor in the Retina

**DOI:** 10.1371/journal.pone.0060633

**Published:** 2013-04-02

**Authors:** Julia Hlawatsch, Marcus Karlstetter, Alexander Aslanidis, Anika Lückoff, Yana Walczak, Michael Plank, Julia Böck, Thomas Langmann

**Affiliations:** 1 Institute of Human Genetics, University of Regensburg, Regensburg, Germany; 2 Department of Ophthalmology, University of Cologne, Cologne, Germany; University of Oldenburg, Germany

## Abstract

Inherited retinal diseases are mainly caused by mutations in genes that are highly expressed in photoreceptors of the retina. The majority of these genes is under the control of the transcription factor Cone rod homeobox (Crx), that acts as a master transcription factor in photoreceptors. Using a genome-wide chromatin immunoprecipitation dataset that highlights all potential *in vivo* targets of Crx, we have identified a novel sterile alpha motif (SAM) domain containing protein, Samd7. mRNA Expression of Samd7 was confined to the late postnatal and adult mouse retina as well as the pineal gland. Using immunohistochemistry and Western blot, we could detect Samd7 protein in the outer nuclear layer of adult mouse retina. Ectopic over-expression in HEK293 cells demonstrated that Samd7 resides in the cytoplasm as well as the nucleus. *In vitro* electroporation of fluorescent reporters into living mouse retinal cultures revealed that transcription of the Samd7 gene depends on evolutionary conserved Crx motifs located in the first intron enhancer. Moreover, Crx knock-down with shRNA strongly reduced Samd7 reporter activity and endogenous Samd7 protein, indicating that Crx is required for retinal expression of Samd7. Finally, using co-transfections in luciferase reporter assays we found that Samd7 interferes with Crx-dependent transcription. Samd7 suppressed luciferase activity from a reporter plasmid with five Crx consensus repeats in a dose dependent manner and reduced Crx-mediated transactivation of regulatory sequences in the retinoschisin gene and the Samd7 gene itself. Taken together, we have identified a novel retinal SAM domain protein, Samd7, which could act as a transcriptional repressor involved in fine-tuning of Crx-regulated gene expression.

## Introduction

Rods and cones of the retina are highly specialized cells required for phototransduction, the biochemical key step of visual perception. Up to now, mutations in more than 170 inherited retinal disease genes have been identified, which often lead to malfunction of retinal cells and progressive retinal degeneration (Retnet database, http:/www.sph.uth.tmc.edu/Retnet/). Within this large group of causative genes, defects in retina-specific genes and key transcription factors are frequently associated with inherited retinal dystrophies [1]. There is a strong correlation between high transcript levels of a gene in photoreceptors and a dysfunction of the corresponding protein, which can subsequently lead to retinal disease [2]. Therefore, the identification of abundantly expressed genes in the retina and knowledge about their regulation may help to find yet unknown genetic causes for retinopathies.

Photoreceptor-specific gene regulation is controlled by a hierarchical network of transcription factors including orthodenticle homeobox 2 (Otx2) [3], cone rod homeobox (Crx) [4], [5], neural retina leucine zipper (Nrl) [6], nuclear receptor subfamily 2 group E member 3 (Nr2e3) [7], thyroid hormone receptor beta 2 (Thrb2) [8], and retinoid related orphan receptor beta (Rorb) [9]. Crx is present in developing as well as adult rod and cone photoreceptors, where it critically influences the transcription of most photoreceptor-specific genes [10]. Accordingly, Crx acts like a classical terminal selector gene, which maintains and controls the terminally differentiated state of rods and cones [11]. ChIP-seq experiments in the mouse retina revealed that Crx coordinates the expression of several hundreds of photoreceptor genes including most retinal disease genes [12]. In a candidate-gene prioritization strategy based on these Crx ChIP-seq data, two novel retinitis pigmentosa genes, FAM161A and MAK, were recently identified [13], [14]. Thus, the Crx ChIP-seq dataset is highly useful to identify novel retina-specific genes and define novel targets for genetic analyses.

Sterile alpha motif (SAM) domains are 70 amino acid long protein-protein interaction domains, which are present in a variety of proteins from different functional classes [15]. These proteins often self-associate via their SAM domains and some form polymeric complexes, which is required for modulation of functional activity [16]. SAM proteins can act as kinases [17], regulatory enzymes [18], scaffolding proteins [19], RNA-binding proteins [20], [21], and transcriptional regulators [22], [23]. The Ets transcription factor Yan contains a SAM domain and is a negative regulator of photoreceptor development [24]. Major retinal SAM domain protein (Mr-s, alias Samd11) was identified as the first SAM domain protein predominantly expressed in rod photoreceptor cells and the pineal gland [25]. Mr-s, which contains an isolated SAM domain, is regulated by Crx and likely functions as a transcriptional repressor involved in photoreceptor development [25].

In this study, we cloned and characterized Samd7, the hitherto second SAM domain containing protein specifically expressed in the mammalian retina and pineal gland. Samd7 is confined to the outer nuclear layer of the developing and adult mouse retina and its transcription is controlled by Crx-bound *cis*-regulatory elements. We found that Samd7 is mainly localized in the nucleus, where it blocks Crx-dependent transcription from retina-specific promoters including those from the retinoschisin gene and the Samd7 gene itself. These findings propose a novel role for Samd7 in transcriptional regulation of retina-specific genes.

## Materials and Methods

### Mouse husbandry

CD1 and C57BL/6 mice were purchased from Charles River Laboratories (Sulzfeld, Germany) and maintained on a 12 hour light/dark schedule at 22°C with free access to water and food. The health of the animals was regularly monitored, and all procedures were approved by the University of Regensburg animal rights committee and complied with the German Law on Animal Protection and the Institute for Laboratory Animal Research Guide for the Care and Use of Laboratory Animals, 1999.

### DNA constructs

Mouse retinal cDNA was used to amplify the full-length Samd7 open reading frame using forward primer 5′-cccaagcttatgacaaacccaatgatgtctgtgag-3′ and reverse primer 5′-cccgaattcttaatttctcataacgtcttgctcag-3′. The PCR-product was cloned into the pFLAG-CMV-4 vector (Sigma Aldrich) at restriction sites *Hind*III and *Eco*RI. The clone was validated by cycle sequencing with vector-specific and internal primers. The pCAG-GFP and No-basal/Rho-basal dsRed vectors have been described previously [12]. The RNAi vector pBS/U6-Crx-shRNA has been described previously [26] and the plasmid pLKO.1 scramble shRNA was purchased from Addgene.

To create the Samd7-CBR1-dsRed construct, PCR was used to amplify a 495 bp region of the mouse Samd7 promoter region using forward primer 5′-tccccggaattcgcccattctcacctagagca-3′ and reverse primer 5′-ttcggggtaccgcccttgctgacagctctt-3′. The promoter fragment was subcloned upstream of dsRed in the no-basal reporter vector using *EcoR*I and *Kpn*I restriction enzymes. The 581 bp insert of the Samd7-CBR2-dsRed construct was amplified from mouse genomic DNA with forward primer 5′-tcccctctagagatgtgggcaactcaaacct-3′ and reverse primer 5′-tccccggaattcagacagccactctggatggt-3′. The intron fragment was subcloned in the Rho-basal reporter vector at *Xba*I and *EcoR*I restriction sites. Site-directed mutagenesis was performed to replace the central core motif of Crx binding sites within Crx-bound region 2 (CBR2) using the QuikChange Multi Site-Directed Mutagenesis Kit (Stratagene, La Jolla, CA, USA). In general, the TAAT site was substituted by CCCC nucleotides. The following primers were used for mutagenesis: CBR2/CBS1, forward 5′-cgtctgaaggcagcgccccaggcatcttcat-3′, reverse 5′-aggatgaagatgcctggggcgctgccttcag-3′, CBR2/CBS2, forward 5′-catcttcatcctgcccccgtgctggccatccag-3′, reverse 5′-ggatggccagcacgggggcaggatgaagatgcc-3′, CBR2/CBS3, forward 5′-ggccatccaggccccgagctctgggtcagag-3′, reverse 5′-acccagagctcggggcctggatggccagcac-3′, CBR2/CBS4, forward 5′-ctctgttttgagccccccagacacactctaca-3′, reverse 5′-gagtgtgtctggggggctcaaaacagagaggg-3′.

To clone the Samd7-CBR1-Luciferase construct, PCR was used to amplify a 495 bp region of the mouse Samd7 promoter region using forward primer 5′-ttcggggtaccgcccattctcacctagagca-3′ and reverse primer 5′-ggtaccgctcgaggcccttgctgacagctctt-3′. The promoter fragment was subcloned upstream of luciferase in the pGL4.10 reporter vector using *Kpn*I and *Xho*I restriction enzymes. The Rs1 luciferase construct has been described previously [27]. Design and cloning of the p5xCrx-tk-Luc plasmid was published recently [28].

### Immunohistochemistry

For retinal immunofluorescence studies, cryo-sections were fixed with 4% paraformaldehyde and rinsed with PBS. Sections were then rehydrated in PBS and preincubated with 1% dried milk in PBS and 0.01% Tween 20 to reduce nonspecific staining. Overnight incubation with the primary anti-Samd7 antibody (Q-12, Sc100141, Santa Cruz Biotechnology, Santa Cruz, CA, USA) was performed at 4°C in PBS containing 2% BSA, 0.02% NaN3 and 0.1% Triton X-100. After washing in PBS, samples were labeled for 1h at room temperature with the secondary goat anti-rabbit antibody conjugated to Alexa594 (red) (Dianova, Hamburg, Germany). Nuclei counter-staining was performed with 0.1 µg/ml 4',6-diamidino-2-phenylindol in PBS (Molecular Probes, Life Technologies, Frankfurt, Germany) for 10 min at room temperature. The cryo-sections were mounted with fluorescent mounting medium (Dako Cytomation, Hamburg, Germany) and viewed with a Zeiss Axio Imager fluorescence microscope equipped with ApoTome.2 (Carl Zeiss, Jena, Germany). Microscopic pictures were analyzed with ZEN software (Carl Zeiss, Jena, Germany).

### Immunocytochemistry

HEK293 cells were seeded on glass coverslips and transfected with the Flag-Samd7 expression plasmid for 48 hours. Slides were fixed with 4% paraformaldehyde, washed with PBS and incubated in blocking buffer containing 10% goat serum and 0.3% Triton X-100. Cells were then incubated with the anti-Samd7 antibody (Q-12, Sc100141, Santa Cruz Biotechnology, Santa Cruz, CA, USA) or the anti-Flag antibody (#2368, Cell Signaling technology, Cambridge, UK) in a solution containing 2.5% goat serum and 0.1% Triton X-100 for 1 hour at room temperature. After 30 min incubation with the secondary antibody conjugated to Alexa594 (red), slides were washed with PBS and counterstained with DAPI in PBS. Labeled cells were viewed with a Zeiss Axio Imager fluorescence microscope equipped with ApoTome.2 (Carl Zeiss, Jena, Germany). Microscopic pictures were analyzed with ZEN software (Carl Zeiss, Jena, Germany).

### Western blot analysis

Mouse retinal tissue was homogenized in cold RIPA buffer (50 mM Tris/HCl pH 7.4, 150 mM NaCl, 1% Triton X-100, 0.5% sodium deoxycholate, 0.1% SDS, and protease inhibitors) using a TissueLyser (Qiagen, Hilden, Germany). Insoluble debris was removed by centrifugation for 5 min at 5000 g. HEK293 cells transfected with Flag-Samd7 and mock vectors were directly lysed in RIPA buffer. Nuclear and cytoplasmic extracts were prepared using the NE-PER nuclear protein extraction kit according to the intructions of the manufacturer (Thermo Scientific, Schwerte, Germany). Protein concentrations were determined by Bradford assay (Roti-quant, Roth, Karlsruhe, Germany). 30 µg of proteins were separated by SDS-PAGE on 10% gels with PageRuler prestained protein ladder (Thermo Scientific, Walthem, MA, USA). Proteins were then transferred to 0.45 µm nitrocellulose membranes (Biorad, Munich, Germany). After blocking in PBS containing 3.5% nonfat dry milk, membranes were incubated with primary antibodies against Samd7 (Q-12, Sc100141, Santa Cruz Biotechnology, Santa Cruz, CA, USA), Flag tag (#2368, Cell Signaling technology, Cambridge, UK), or beta-actin (mAbcam 8224, Cambridge, UK). Blots were then incubated with secondary goat anti-rabbit IgG antibodies conjugated to horseradish peroxidase. Western blot signals were visualized with the Multiimage II system (Alpha Innotech, Santa Clara, CA, USA).

### *In vitro* electroporation and culture of explanted retinas

*In vitro* electroporation of explanted retinas was performed as described previously [12]. Briefly, retinas from P0 wild-type CD1 mouse pups were dissected and placed into a microslide chamber containing a mixture of pCAG-GFP as loading control and the reporter construct driving dsRed expression. Retinas were subjected to five 30 V pulses with 50 ms in length and 950 ms apart using an ECM 830 square-wave electroporator (BTX Harvard Apparatus, Holliston, MA, USA). Each construct was electroporated into three different retinas and three independent electroporations were performed. The retinas were then rinsed in medium and placed on circular Nucleopore filters (25 mm, 0.2 mm; VWR, Darmstadt, Germany) with the lens facing the membrane. After eight days of *in vitro* culture, retinas were fixed and imaged in both flat-mounts and cross-sections. For quantification of promoter activities, dsRed fluorescence was determined and normalized to GFP control fluorescence as described before [29]. Briefly, retinal flat-mount images were captured for the red and the green channels using a fluorescence microscope (Axioskop2 MOT Plus, Zeiss, Jena, Germany). Five regions of interest within each retina and three regions outside each retina were defined using ImageJ software (National Institutes of Health, Bethesda, http://rsbweb.nih.gov/ij/). Then, the mean background-subtracted pixel intensity of the experimental red channel was divided by the mean pixel intensity of the green control channel for normalization.

### Transient transfections, luciferase and beta-Gal assays

HEK293 cells were cultured in DMEM containing 10% FCS, 100 U/ml penicillin/streptomycin at 37°C in a 5% CO_2_ atmosphere. Cells in 12-well plates were transfected with 0.2 µg of reporter plasmid using the TransIT-LT1 Transfection Reagent (Mirus, Madison, WI, USA) following the manufacturer’s instructions. For co-transfections, 0.2 µg luciferase plasmid were used together with various concentrations of pcDNA4/HisMax-Crx, pcDNA4/-Samd7, or empty expression vector. 0.4 µg pSV beta-galactosidase vector (Promega) were co-transfected in each reaction to control for transfection efficiency. Cells were harvested 24h after transfection by scraping in 1x lysis buffer (Promega, Madison, WI, USA). For luciferase assays, 20 µl of cytosolic extract and 100 µl of luciferase assay reagent were mixed and light emission was measured with a Tecan Infinite F200 pro reader (Tecan, Crailsheim, Germany). All data were normalized for beta-galactosidase activity using the Promega beta-galactosidase enzyme assay (Promega), calculating the absorbance at 420 nm. Fold activation was calculated relative to control transfected cells. For each construct, at least six independent experiments were performed. Statistical significance was determined using One-way Analysis of Variance and Tukey's Post Hoc Test.

### RNA-Isolation, RT-PCR and quantitative (real-time) RT-PCR

Total RNA was isolated from different mouse tissues and retinas at different postnatal ages using the RNeasy Mini Kit (Qiagen, Hilden, Germany). RNA quality was assessed on the Agilent 2100 Bioanalyzer with the RNA 6000 Nano LabChip reagent kit (Agilent Technologies, Palo Alto, CA). Reverse transcription was performed using the RevertAid H Minus First Strand cDNA Synthesis kit (Fermentas, St. Leon-Rot, Germany).

RT-PCR to amplify 563 bp of mouse Samd7 from stomach, lung, liver, testis, kidney, spleen, brain, retina, heart, muscle, and pineal gland was performed with 50 ng cDNA and primers forward, 5′-tcacttctactcaggctggggca-3′and reverse, 5′-gttctccgtgggggttggcg-3′. A 293 bp product of Samd11 was amplified from pineal gland cDNA using primers forward, 5′-tgtccagcccagccaacccaag-3′ and reverse, 5′-tgtggtctcctcatcagtgaaga-3′. A 292 bp fragment of β-actin was amplified as reference with primers forward, 5′-acccacactgtgcccatcta-3′ and reverse, 5′-cggaaccgctcattgcc-3′ using the Taq Core kit (Qiagen, Hilden, Germany) and standard PCR conditions with 25 cycles.

qRT-PCR was carried out with the TaqMan 7900HT PCR detection system (Invitrogen Life Technologies, San Diego, CA) in 10 µl reaction mixture containing 1x TaqMan Gene Expression Master Mix (Invitrogen Life Technologies), 200 nM primers and 0.25 µl dual-labeled probe (Roche Universal Probe Library, Roche Diagnostics, Mannheim, Germany). For the detection of mouse Samd7 transcripts, intron-spanning primers forward, 5′-tgatggaaagaatggggttt-3′ and reverse, 5′-tctgagtgcaacctgctcat-3′ were combined with universal probe # 34. Atp5b was amplified as stable reference gene using primers forward, 5′-ggcacaatgcaggaaagg-3′ and reverse, 5′-tcagcaggcacatagatagcc-3′ together with probe # 77. The PCR reaction parameters were as follows: 40 s at 95°C melting, 1 min at 60°C annealing, and 2 min at 72°C extension. Each run was performed for 40 cycles and each measurement was performed in biological triplicates. PCR efficiencies of both products were determined with serial dilutions of mouse retinal cDNA and were shown to be >90% (Figure S1). Results were analyzed with the ABI sequence detector software version 2.3 using the ΔΔCt method for relative quantitation.

## Results

### Cloning of mouse Samd7

**Figure 1:**
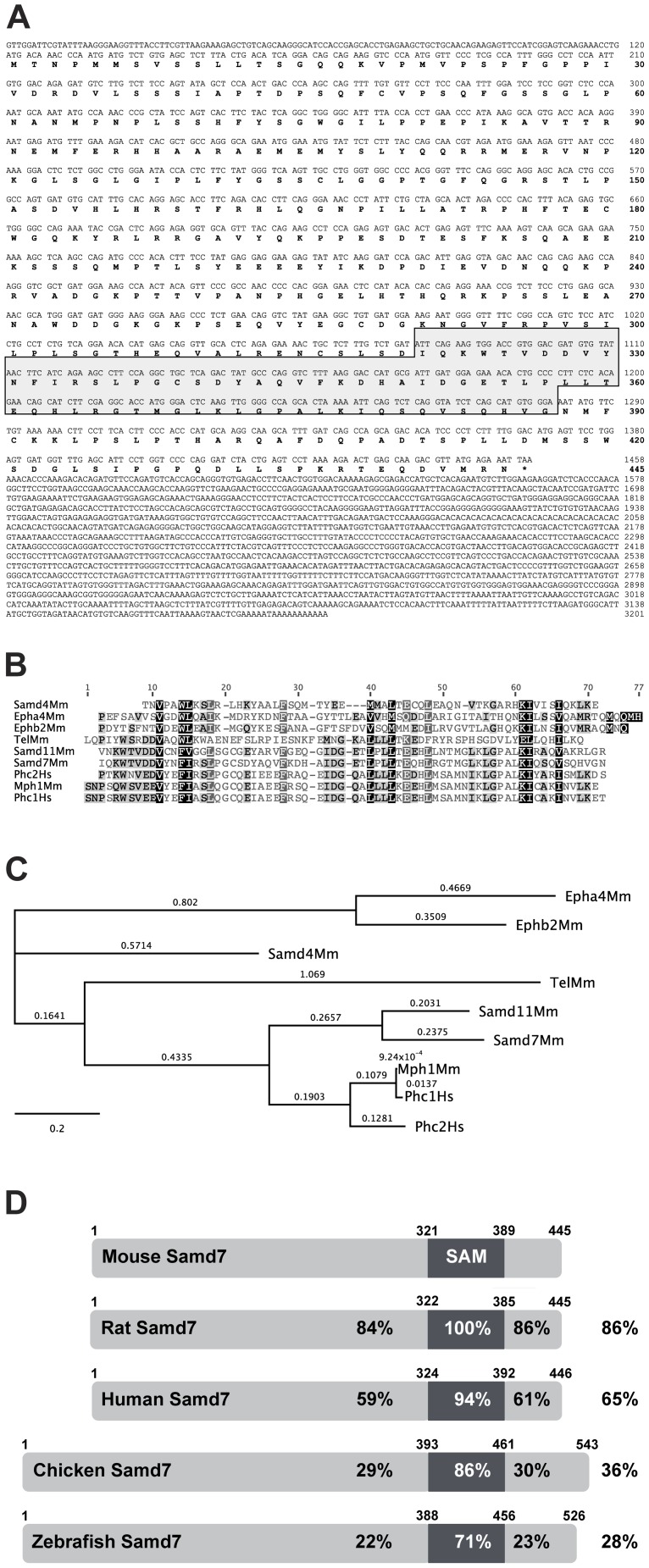
Samd7 is a novel phylogenetically conserved SAM-domain protein. A: The full-length Samd7 protein comprises 445 amino acids including a 67 aa SAM domain, which is indicated by a box. B: Amino acid alignment of selected SAM domain sequences using Clustal W Blosum 62. The level of similarity is indicated by shading ranging from 100% (black) to less than 60% (light gray). C: Phylogenetic conservation of SAM-domain containing proteins. The branch length represents the number of substitutions that have ocurred in that branch and the distance scale represents the number of differences between sequences, with 0.1 meaning 10% difference between two sequences. D: Amino acid sequence similarities of mouse, rat, human, chicken, and zebrafish Samd7 proteins. The percentage of similarity is shown for the full-length protein as well as for individual regions of the protein, respectively.

We have recently analyzed the targetome of the retinal transcription factor Crx using chromatin immunoprecipitation with massively parallel sequencing (ChIP-seq) [12]. Using this dataset, we have now identified a previously uncharacterized SAM domain containing protein, Samd7. In the ChIP-seq dataset, Samd7 was the gene locus with most Crx ChIP-seq reads (data not shown), indicating high expression and a putative important function in the mouse retina. To clone the 445 amino acid open reading frame (ORF) of the mouse Samd7 gene, we carried out RT-PCR with cDNA from 2 month old mouse retina. The Samd7 ORF contains an isolated SAM domain in the C-terminal part (Figure 1A), which exhibits high homology with SAM domains of the known proteins Samd4, Epha4, Ephb2, Tel, Samd11, Phc2, Mph1, and Phc1 (Figure 1B). Phylogenetic analysis indicated that the closest relative of Samd7 is Samd11, which also lacks further known protein domains (Figure 1C). Samd11 has been recently characterized as *major retinal SAM domain protein* (Mr-s), which may function as a transcriptional repressor in photoreceptor cells [25]. The mouse Samd7 ORF and especially its SAM domain is highly conserved in rat, human, chicken and zebrafish (Figure 1D). Samd7 maps to mouse chromosome 3A3. The human ortholog resides at 3q26.2, which does not harbor a retinal disease candidate locus so far.

### Samd7 is expressed in the mouse retina and pineal gland

**Figure 2:**
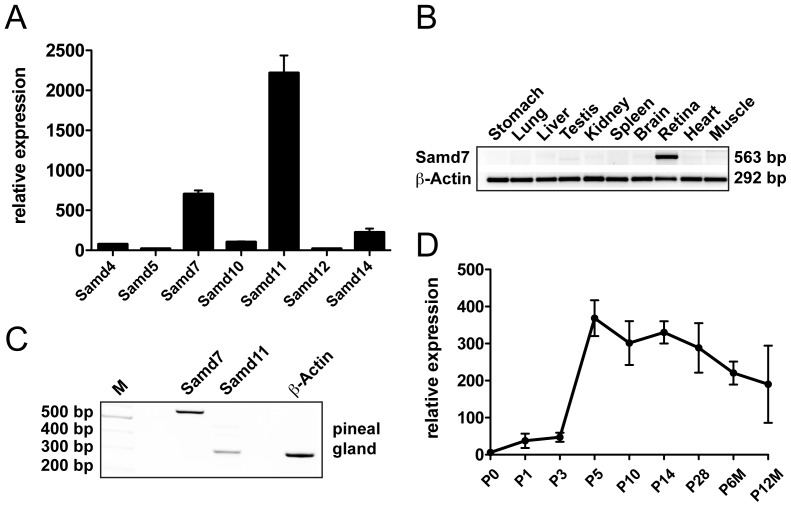
Samd7 is expressed in the mouse retina and pineal gland. A: Relative mRNA expression of all SAM-domain containing proteins with isolated SAM domains in postnatal day 7 mouse retinas. Mean signal intensities of three independent Affymetrix mouse expression 430A arrays (GEO accession number GSE5581) show that Samd7 is the second most abundant transcript in the retina. B: RT-PCR analysis of total RNAs extracted from mouse stomach, lung, liver, testis, kidney, spleen, brain, retina, heart, and muscle reveals retina-specific mRNA expression of Samd7. C: RT-PCR analysis of total RNA extracted from mouse pineal gland. Primer pairs specific for Samd7, Samd11 and beta-actin were used for PCR. D: Real-time qRT-PCR analysis of early postnatal and adult retina demonstrates that Samd7 expression peaks at postnatal day 5 and then stays at intermediate levels.

To compare retinal mRNA expression of Samd7 with other SAM domain proteins that lack additional functional domains, a DNA-microarray dataset that was previously published by our group was screened [30]. Seven SAM domain only proteins were present on the microarray and Samd11 (alias Mr-s) showed highest expression levels in the postnatal day (P) 7 retina (Figure 2A). Samd7 and Samd14 were also significantly expressed in the retina, whereas Samd4, Samd5, Samd10, and Samd12 were only weakly expressed (Figure 2A). To analyze the tissue specificity of Samd7 expression, transcript levels were amplified in various adult mouse tissues using RT-PCR. We observed that Samd7 was highly expressed in the retina but was not found in stomach, lung, liver, testis, kidney, spleen, brain, heart, or muscle (Figure 2B). As many photoreceptor-specific genes, including the related Samd11 gene, are expressed in the pineal gland [25], [31], we next analyzed Samd7 transcripts in this tissue. RT-PCR amplification verified the previously described weak Samd11 expression [25] and showed a strong band specific for Samd7 (Figure 2C). We then investigated the temporal expression of Samd7 mRNA in the late stages of mouse retinal development using real-time qRT-PCR. Weak transcript levels were detected at birth and between P1 and P3 (Figure 2D). At P5, Samd7 showed a peak of expression, which slowly declined to intermediate levels at higher mouse ages (Figure 2D). The early expression pattern of Samd7 clearly parallels the maturation of photoreceptors and we thus speculate that the protein functions mainly in terminally differentiated photoreceptors.

### Samd7 is localized to the outer nuclear layer of the retina and resides in the cytoplasm and nucleus of transfected cells

**Figure 3:**
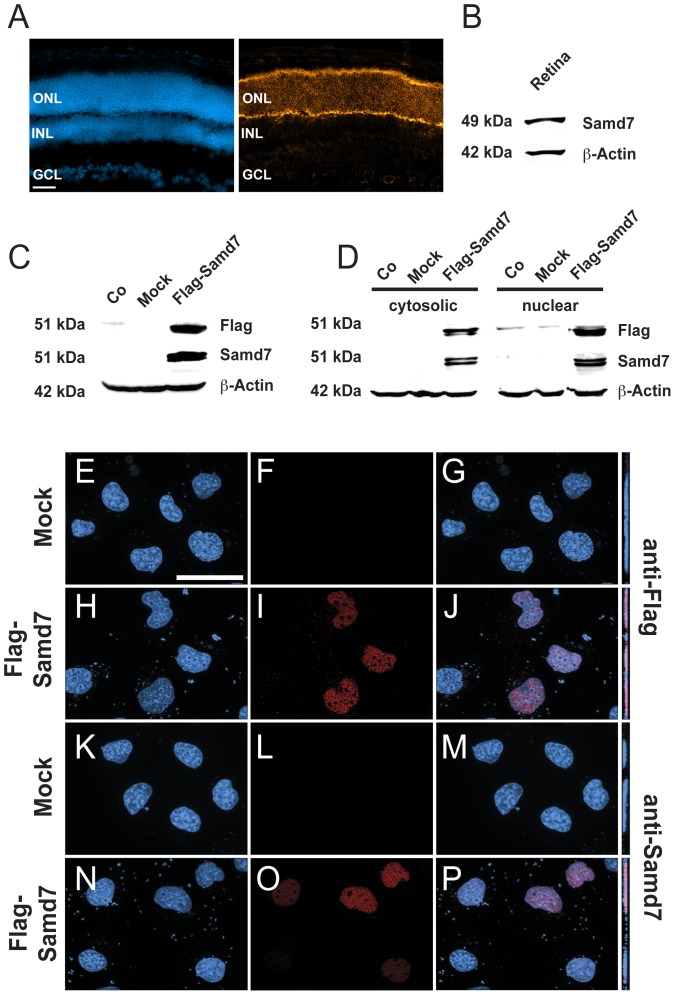
Samd7 is expressed in the outer nuclear layer of the mouse retina and localizes to the nucleus of transfected cells. A: Immunhohistochemical analysis shows that Samd7 localizes to the outer nuclear layer in the adult mouse retina. Left panel: DAPI staining, right panel: anti-Samd7 antibody staining, ONL: outer nuclear layer, INL: inner nuclear layer, GCL: ganglion cell layer. Scale bar, 50 µm. B: Western blot performed with retinal lysates detecting Samd7 at a molecular weight of 49 kDa and beta-actin as loading control. C: Western blot performed with protein lysates from naive HEK293 cells (Co) or HEK293 cells transfected with mock plasmid or Flag-Samd7 expression plasmid. Anti-Samd7 antibody, anti-flag antibody, and anti-beta-actin antibody were used. The Flag-Samd7 band had a molecular weight of approximately 51 kDa. D: Western blot of cytosolic and nuclear fractions of HEK293 cells transfected with mock plasmid or Flag-Samd7 expression vector. Samd7 was was detected in both the cytosolic and nuclear fractions at a molecular weight of 51 kDa. (E–P): Subcellular localization of Samd7 in HEK293 cells transfected with Flag-Samd7 expression vector shown in fluorescent Z-stacked optical images. Mock transfected cells did not show a specific red signal with either the anti-Flag (F) or the anti-Samd7 (L) antibody. The anti-Flag antibody (I, J) as well as the anti-Samd7 antibody (O, P) showed a specific nuclear staining in Flag-Samd7 transfected cells when couter-stained with DAPI.

Given the high mRNA expression in the retina, our next goal was to determine the localization of the Samd7 protein in the mouse retina. We first performed immunohistochemistry of adult mouse retinal sections using a commercial anti-Samd7 antibody. These experiments showed that Samd7 was predominantly expressed in the outer nuclear layer, where rod and cone photoreceptors reside (Figure 3A). We then performed Western blot analysis to confirm specificity of the antibody. The 49 kDa Samd7 protein was detected as a specific band in retinal extracts from adult mice (Figure 3B). To further corroborate antibody specificity, we cloned and expressed a recombinant Flag-tagged Samd7 protein in HEK293 cells. A specific band of 51 kDa was detected with both, the anti-Flag antibody and the anti-Samd7 antibody (Figure 3C). This result confirms that the anti-Samd7 antibody specifically recognizes Samd7.

To study the subcellular localization of Samd7 in mammalian cells, we again analyzed HEK293 cells transiently transfected with a full-length Flag-tagged Samd7 expression construct. Cell lysates were separated into cytoplasmic and nuclear fractions and Western blots were performed. Using the anti-Flag and the anti-Samd7 antibody, corresponding Samd7 bands were identified in the cytoplasm as well as the nuclear fractions. Stronger signals were detected in the nuclear fractions which were often seen as double bands with slightly different molecular weights at an approximate size of 51 kDa (Figure 3D). We next analyzed mock-transfected and Samd7 transfected cells with immunocytochemistry using anti-Flag and anti-Samd7 antibodies (Figure 3E-P). Fluorescence microscopy of immunostained cells which were counter-stained with DAPI showed a predominant nuclear localization of Samd7 (Figure 3H–J, N–P). These data suggest that a significant portion of Samd7 protein resides in the nucleus. Interestingly, its closest relative Samd11 also shows a predominant distribution in the nucleus when transfected into HEK293 cells [25].

### Samd7 transcription is controlled by Crx

**Figure 4:**
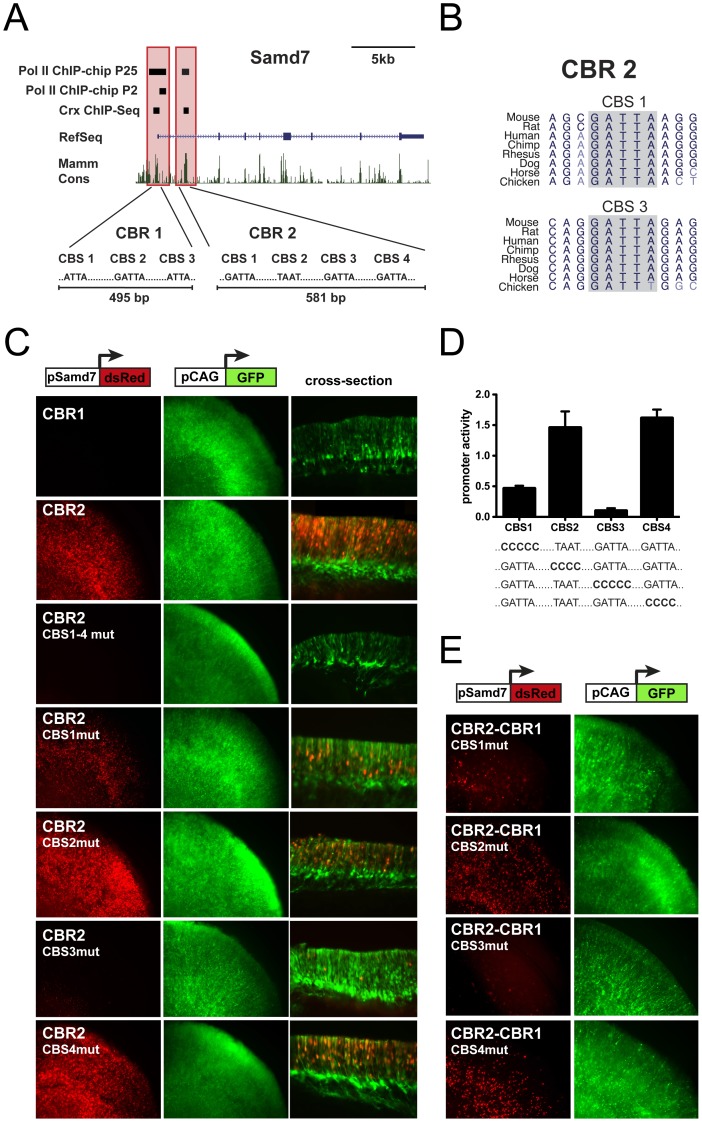
Samd7 transcription is regulated by Crx. A: Identification of two Crx-bound regions (CBR1 and CBR2) at the mouse Samd7 locus. Enriched Crx ChIP-seq regions are shown in the proximal promoter and first intron of Samd7 [12]. RNA polymerase II ChIP-Chip peaks at P2 and P25 [32] overlap with the Crx ChIP-Seq regions. The degree of mammalian conservation is indicated at the bottom. The nucleotide sequences of canonical Crx binding sequences (CBS) within CBR1 and CBR2 are depicted. B: The phylogenetic conservation of CBS1 and CBS2 within CBR2 is shown for several species. C-E: Activity of wild-type and mutant Samd7 CBRs in explanted mouse retinas. Co-electroporations were performed with pCAG-eGFP as control and the indicated Samd7 regulatory elements fused to dsRed. All constructs were electroporated at postnatal day 0 and the cultured explants were harvested at postnatal day 8. C: CBR1 is not active when fused to a promoterless dsRed reporter cassette. In contrast, CBR2 drives strong dsRed expression when fused to the minimal Rhodopsin promoter, which is *per se* not active. The cross-sections show that dsRed signals driven by Samd7 CBR2 were localized in the ONL, whereas the GFP signals by the ubiquitous control promoter were localized in the ONL, INL and GCL. ONL: outer nuclear layer, INL: inner nuclear layer, GCL: ganglion cell layer. D: Quantitative analysis of mutant constructs demonstrates that CBS1 and CBS3 are mandatory for high reporter expression of the intronic CBR2. E: Enhancer activity of CBR2 upstream of CBR1 also requires intact nucleotide sequences at CBS1 and CBS3.

The identification of Samd7 as *in vivo* target of Crx in the Crx ChIP-seq study [12] and its retina-specific expression suggests that Samd7 transcription is directly regulated by Crx. We inspected the location of retinal Crx ChIP-seq reads at the Samd7 locus and identified two Crx-bound regions (CBRs) in the promoter region and the first intron, respectively (Figure 4A). Sequence analysis of a recently published RNA Polymerase II ChIP-chip dataset [32] also showed a significant Pol II association with the promoter region of Samd7 at postnatal day 2 (Figure 4A). At postnatal day 25, when the retina is fully developed, Pol II peaks were identified at the promoter as well as in the first intron. These Pol II bound regions fully overlap with both CBRs (Figure 4A), indicating that these regulatory sites indeed function as initiation and elongation sites of Samd7 transcription in the adult retina.

Bioinformatic sequence analysis using MatInspector then showed that CBR1 contains three canonical Crx binding sequences (CBS1–3), whereas CBR2 contains four CBS (Figure 4A). A nearly perfect phylogenetic conservation of Crx sites was found in CBS1 and CBS3 residing within CBR2 (Figure 4B). To test whether CBR1 and CBR2 represent active *cis*-regulatory regions, CBR-dsRed reporter fusions were electroporated into living mouse retinas. CBR1 located in the proximal promoter region of the Samd7 gene failed to drive detectable dsRed expression in the retina (Figure 4C). In contrast, intronic CBR2 upstream of a minimal rhodopsin promoter, which by itself is not active, drove strong expression with a predominant fluorescence signal in the outer nuclear layer, where photoreceptors are localized (Figure 4C). To evaluate the contribution of individual Crx binding sites to the strong activity of CBR2, site-directed mutagenesis was performed in the critical Crx core motifs. Mutations in all four CBS of CBR2 simultaneously resulted in a complete loss of activity in electroporated retinal explants (Figure 4C). Electroporations with four constructs that eliminated each CBS independently were then performed and fluorescence levels were quantified. Mutation of CBS1 and CBS3 nearly abolished enhancer activity, whereas mutagenesis of CBS2 and CBS4 had no major effect (Figure 4D). To analyze whether the same sites of intronic CBR2 also enhance activity of its own promoter, mutant CBR2 fragments were cloned upstream of CBR1 and the reporter activity was determined. In accordance with the data from CBR2 electroporations alone, mutagenesis of CBS1 and CBS3 reduced dsRed expression, whereas nucleotide changes in CBS2 and CBS4 had no effect on reporter activity (Figure 4E). Thus, CBS1 and CBS3 in the intronic enhancer CBR2 are indispensable for Crx-regulated expression of Samd7 in the retina.

**Figure 5:**
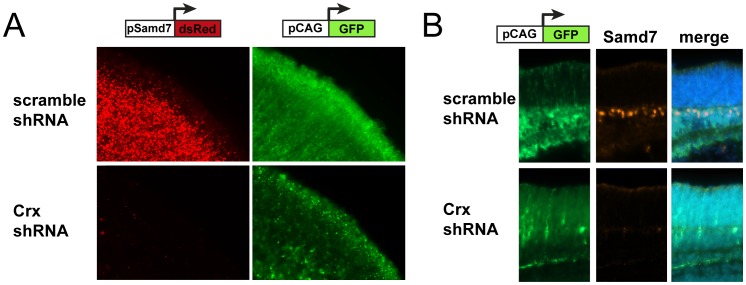
Samd7 expression is reduced in Crx knock-down retinas. A: Activity of wild-type Samd7 CBR2 fused to dsRed was strongly reduced in explanted P8 mouse retinas co-electroporated with Crx shRNA. B: Reduced staining of endogenous Samd7 with anti-Samd7 antibody in P8 mouse retinas electroporated with Crx shRNA compared to scamble shRNA control. pCAG-eGFP was used as electroporation control and scrable shRNA vector served as negative control for knock-down experiments. DAPI staining was performed in merged images.

To further examine whether Crx is essential for Samd7 gene activity, we performed Crx knock-down experiments in explanted mouse retinas. Therefore, electroporations of Crx shRNA plasmids or scrambled shRNA negative controls together with the Samd7 CBR2 reporter construct were carried out. These experiments revealed a complete loss of dsRed fluorescence in flat-mount Crx knock-down retinas compared to scrambled shRNA retinas (Figure 5A). This indicates that the Crx-bound sequences in CBR2 are critically dependent on Crx. As a next step, we investigated endogenous Samd7 protein expression in the same Crx knock-down system using immunohistochemistry. In the postnatal day 8 retina co-electroporated with scrambled shRNA control, Samd7 protein was present at the border of the inner nuclear layer and the outer nuclear layer (Figure 5B, upper panel). This specific staining of Samd7 almost completely disappeared in Crx knock-down retinas (Figure 5B, lower panel), suggesting that endogenous Samd7 expression requires the presence of Crx.

### Samd7 functions as a transcriptional repressor

**Figure 6:**
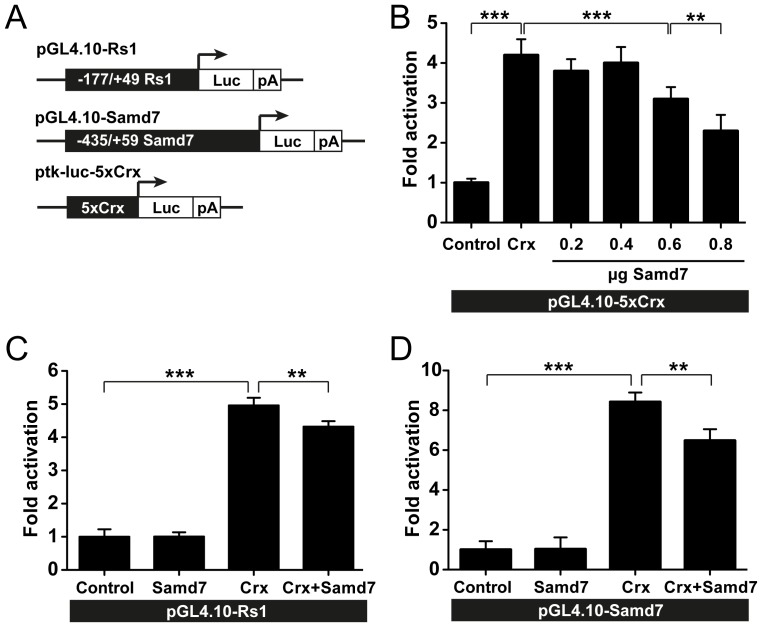
Samd7-dependent suppression of Crx-activated promoters. A: Schematic drawing of retinoschisin, Samd7, and 5xCrx tk Luc reporter constructs used for luciferase assays in HEK293 cells. B: The 5xCrx-tk-Luc construct was co-transfected with Crx and various concentrations of Samd7 expression plasmids. C: Samd7 expression plasmid was co-transfected in the absence or presence of Crx vector and the retinoschisin promoter activity was determined. D: Samd7 expression plasmid was co-transfected in the absence or presence of Crx vector and the Samd7 promoter activity was determined. pSV β-galactosidase vector was co-transfected in each reaction to control for transfection efficiency. Error bars represent standard deviation of the mean (n = 6). **P<0.01 and ***P<0.001 One-way Analysis of Variance and Tukey's Post Hoc Test.

Samd7 is localized in the ONL of the adult retina and is present in the nucleus of transfected cells. Thus, we hypothesized that the protein could be a transcriptional regulator. In the absence of an obvious DNA-binding domain, we speculated that Samd7 may interfere with the activity of retinal transcription factors like Crx. To test this hypothesis, the effect of Samd7 co-transfection on Crx-dependent promoter activity was studied with luciferase reporter assays. In the first series of experiments, we used a previously published Crx-dependent luciferase reporter which contains five repeats of Crx consensus sites under the control of a thymidine kinase minimal promoter [28]. As expected, this construct showed a five-fold increase when Crx was co-transfected (Figure 6A). Samd7 exerted a significant dose-dependent suppressive effect on this universal Crx reporter construct (Figure 6B). We selected the most effective concentration from these titration experiments and then analyzed specific regulatory sequences. The retinal expression of the murine and human retinoschisin (RS1) gene is under the control of Crx and thus represents a bona fide Crx target gene [27]. Therefore, this promoter was selected for Crx-specific transactivation assays in the absence or presence of Samd7 expression plasmid (Figure 6C). We could confirm the results from the universal 5xCrx-tk-Luc construct and showed that Crx co-transfection strongly increased luciferase activity of a RS1 reporter construct in HEK293 cells (Figure 6C). The increased luciferase level controlled by Crx was significantly suppressed by Samd7 (Figure 6C). As the Samd7 gene itself is regulated by Crx, we next studied the effects of Samd7 co-transfection on its own promoter construct. As expected, Crx-transfection markedly increased luciferase activity of the proximal Samd7 promoter (Figure 6D). In analogy to the retinoschisin gene, Samd7 co-transfection strongly diminished reporter activity of the Samd7 gene itself (Figure 6D). These experiments suggest that Samd7 functions as a negative regulator of Crx-controlled photoreceptor-specific gene expression.

## Discussion

In this study, we have cloned a novel SAM domain protein, Samd7, which is expressed in the retina and the pineal gland. The peak level of Samd7 expression is at P5, when photoreceptor cells differentiate into their functional form. Immunohistochemistry of mouse retinal sections showed that the Samd7 protein is mainly present in the outer nuclear layer. Ectopic expression of Samd7 in cultured cells revealed a distribution in the cytoplasm and the nucleus. The retinal expression and *cis-*regulatory activity of the Samd7 locus is critically controlled by a phylogenetically conserved Crx-bound enhancer region in the first intron as shown by explant electroporation and knock-down experiments. The potential function of Samd7 was further evaluated in luciferase co-transfection assays. These experiments showed that Samd7 inhibits synthetic Crx-regulatory sequences and specific promoter activities of Crx target genes and thus may function as a novel transcriptional regulator in the retina.

A major conclusion from our mRNA expression data is that Samd7 is now a second SAM domain protein with high expression levels in the retina and the pineal gland. The previously described Mr-s protein (alias Samd11) is the closest phylogenetic relative of Samd7 with a very similar expression profile [25]. Mr-s molecules can self-associate and share a relatively similar protein and SAM domain structure with Samd7 [25]. It is currently not possible to assign interactions or functions to uncharacterized SAM domains via simple computational approaches [33]. However, both Samd7 and Samd11 have an isolated SAM domain in their C-terminal part and lack further known motifs. All other family members with isolated SAM domains, namely Samd1, Samd4, Samd5, Samd10, Samd12, and Samd14 are very weakly expressed in the retina and thus are unlikely to interact with Samd7 or Samd11. Therefore, it will be very interesting to determine in the future whether the retina-specific Samd7 and Samd11 proteins can interact with each other.

Crx-dependent regulation of Samd7 in the mouse retina has been implicated by a previous ChIP-seq study, which showed two significantly enriched Crx peaks around the Samd7 locus [12]. We now could pinpoint the two relevant *cis*-regulatory regions in the promoter and the first intron of the gene using *in vitro* electroporation of reporters into mouse retinas. CBR1, which contains three Crx sites was not active alone and required interaction with the intronic enhancer elements of CBR2 to drive dsRed reporter expression. Mutagenesis of individual Crx motifs in CBR2 revealed two critical binding sites, CBS1 and CBS3, which are almost perfectly conserved among various species. The GATTA core sequence of CBS1 and CBS3 also represents a perfect matrix as deduced from bioinformatic prediction and sequence analysis of more than 5000 Crx ChIP-seq regions [10], [12]. In accordance with this, the previously published Pol II-ChIP-chip dataset revealed that the promoter region and first intron of Samd7 are actively bound by RNA-polymerase II complexes and thus initiate transcription in the adult retina [32]. Our findings define Samd7 as a bona fide Crx-regulated target gene and hence corroborate the assumption that many photoreceptor genes are surrounded by a spatially distributed network of CBRs [34]. Crx often co-regulates photoreceptor genes together with Nrl [35]. However, inspection of published datasets of Nrl^-/-^ retinas revealed no aberrant expression of Samd7 [36]. In contrast, expression profiling of the Otx2-deficient retina revealed a significant down-regulation of Samd7 mRNA levels at P12 [37]. It remains to be explored whether Samd7 is indeed a direct Otx2 target gene, or, whether the loss of Samd7 expression may have been indirectly caused by a reduced Crx expression in Otx2^-/-^ retinas.

Its site of expression in the retina, the nuclear localization, and the domain features shared with the Mr-s protein indicated that Samd7 could be a transcriptional regulator. Indeed, our *in vitro* assays showed that Samd7 interferes with Crx-mediated gene expression at synthetic consensus Crx sites and at two different retina-specific promoters. It is noteworthy that Samd7 exerted this effect without an obvious DNA binding domain. The *Drosophila* SAM protein Mae also contains solely a SAM domain and lacks DNA-binding activity [38]. Mae interacts with and facilitates phosphorylation of the transcriptional repressor Yan via direct interaction with its SAM domain [39]. Phosphorylation of Yan then results in abrogation of its repressor function and induces translocation to the cytoplasm [39]. Therefore, it is possible that Samd7 elicits its repressor function by sequestration of Crx to non-active protein complexes. *In vitro* interaction studies like pull-down assays could further clarify this question. Another possibility could be that Samd7 is part of silencing complexes involving chromatin remodeling enzymes such as histone deacetylases (HDACs). Reporter assays in the presence or absence of HDAC inhibitors like trichostatin A could help to elucidate this mechanism of repression. Samd7 could also act independently from HDACs like the SAM domain containing polycomb complex PRC2, which functions as a methyl transferase and organizes chromatin loops at target genes in their repressed states [40].

Although we have shown that Samd7 can repress transcription from Crx-dependent promoters, the *in vivo* targets of Samd7 are currently unknown. It is obvious that fine-tuning of gene expression in the retina involves activating transcription factors like Crx. However, the role of transcriptional repressors in the control of this Crx-dominated gene regulatory network is poorly understood. Recently, Panky, a photoreceptor-specific ankyrin repeat protein was identified as another transcriptional cofactor that suppresses Crx-regulated photoreceptor genes [28]. We speculate that Samd7 could have a similar function but future studies will be required to precisely clarify the role of Samd7 in retinal gene expression. Mice with targeted disruption or knock-down experiments via *in vivo* electroporation of mouse retinas will be especially helpful to answer these questions.

## Supporting Information

Figure S1 **PCR efficiencies of Samd7 and Atp5b real-time qRT-PCR amplifications.**
(TIF)
