# Supplementary figures and images for: Sterile Alpha Motif Containing 7 (Samd7) Is a Novel Crx-Regulated Transcriptional Repressor in the Retina
Source: PLoS One. 2013 Apr 2;8(4):e60633. doi: 10.1371/journal.pone.0060633 (PMC3615016; doi:10.1371/journal.pone.0060633)

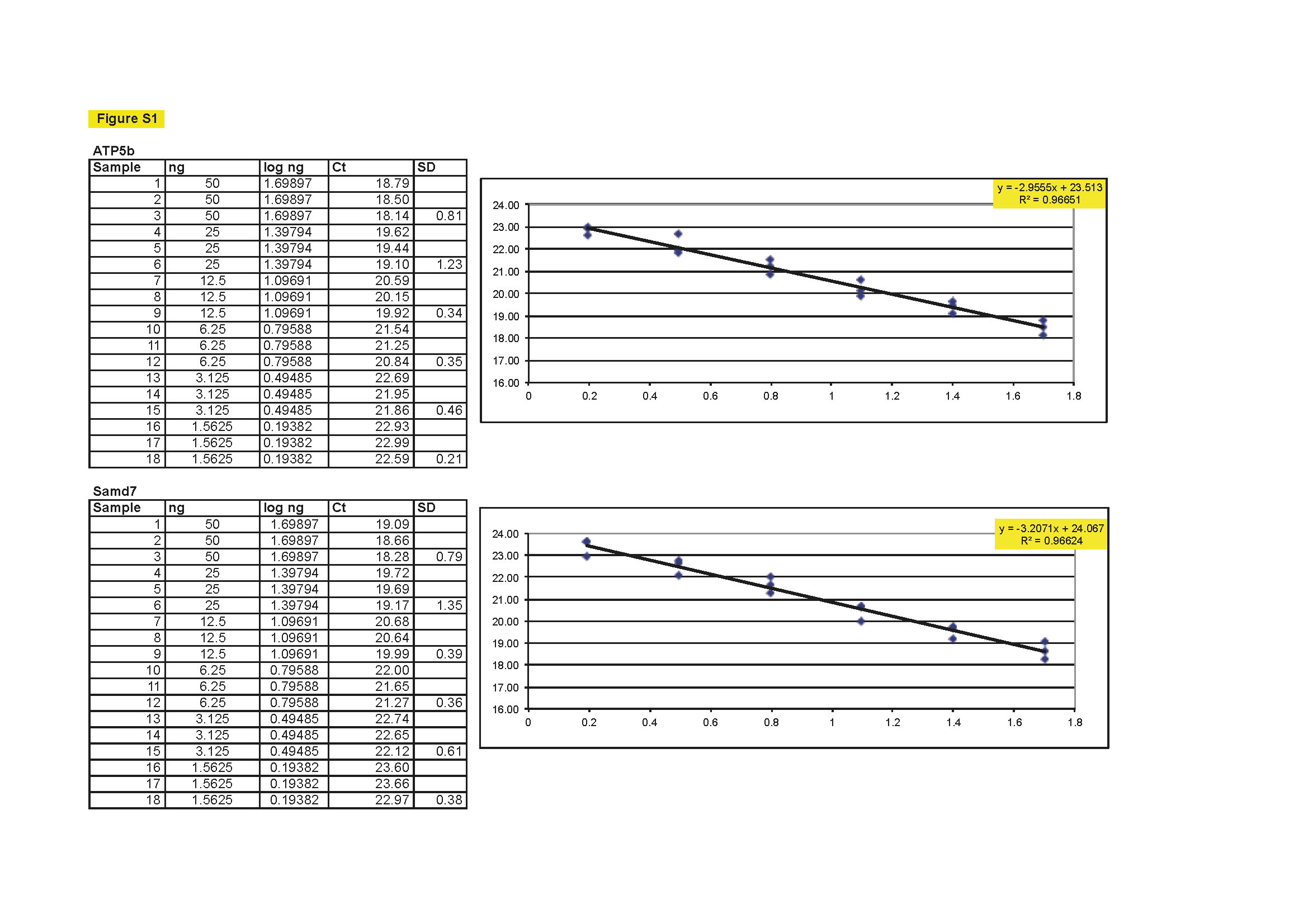

Supplement: Figure S1 — PCR efficiencies of Samd7 and Atp5b real-time qRT-PCR amplifications. (TIF) [file pone.0060633.s001.tif]
